# Distinctive probiotic features share common TLR2‐dependent signalling in intestinal epithelial cells

**DOI:** 10.1111/cmi.13264

**Published:** 2020-10-01

**Authors:** Diana Paveljšek, Karolina Ivičak‐Kocjan, Primož Treven, Mojca Benčina, Roman Jerala, Irena Rogelj

**Affiliations:** ^1^ Biotechnical Faculty Department of Animal Science, University of Ljubljana Domžale Slovenia; ^2^ Department of Synthetic Biology and Immunology National Institute of Chemistry Ljubljana Slovenia; ^3^ Present address: Lek d. d., Verovškova 57, 1526 Ljubljana Slovenia

**Keywords:** *Bifidobacterium*, innate immunity, *Lactobacillus*, postbiotics, TLR10

## Abstract

The underlying mechanisms of probiotics and postbiotics are not well understood, but it is known that both affect the adaptive and innate immune responses. In addition, there is a growing concept that some probiotic strains have common core mechanisms that provide certain health benefits. Here, we aimed to elucidate the signalization of the probiotic bacterial strains *Lactobacillus paragasseri* K7, *Limosilactobacillus fermentum* L930BB, *Bifidobacterium animalis* subsp. *animalis* IM386 and *Lactiplantibacillus plantarum* WCFS1. We showed in in vitro experiments that the tested probiotics exhibit common TLR2‐ and TLR10‐dependent downstream signalling cascades involving inhibition of NF‐κB signal transduction. Under inflammatory conditions, the probiotics activated phosphatidylinositol 3‐kinase (PI3K)/Akt anti‐apoptotic pathways and protein kinase C (PKC)‐dependent pathways, which led to regulation of the actin cytoskeleton and tight junctions. These pathways contribute to the regeneration of the intestinal epithelium and modulation of the mucosal immune system, which, together with the inhibition of canonical TLR signalling, promote general immune tolerance. With this study we identified shared probiotic mechanisms and were the first to pinpoint the role of anti‐inflammatory probiotic signalling through TLR10.

**Abbreviations**
Akt: protein kinase B
APCs: antigen‐presenting cells
IEC: intestinal epithelial cell
IM386: Bifidobacterium animalis subsp. animalis IM386
JAM: junction adhesion molecule
K7: Lactobacillus paragasseri K7
L930BB: Limosilactobacillus fermentum L930BB
MAMP: microbe‐associated molecular patterns
MAPK: mitogen‐activated protein kinase
NF‐κB: nuclear factor kappa B
PAM2: Pam2Cys‐SK4
PAM3: Pam3Cys‐SK4
PI3K: phosphatidylinositol 3‐kinase
PKC: protein kinase C
PKCinh: PKC inhibitor Gӧ6983
PRRs: pattern recognition receptors
TLR: Toll‐like receptor
WCFS1: Lactiplantibacillus plantarum WCFS1
ZO‐1: zonula occludens‐1

## 1 INTRODUCTION

The intestinal epithelial layer is composed of different cell types, which together form a physiological barrier where nutrient metabolism and absorption take place. In addition, epithelial cells are also an important first line of defence, as they are in constant interaction with bacteria and bacterial components on the apical surface and are in close proximity to immune cells on the basolateral side (Goto, 2019). The intestinal epithelium thus serves as a dynamic barrier that is regulated by the immune system through a complex combination of responses involving both innate and adaptive immunity (Allaire et al., 2018). Over the years, research studies have shown that gut microbiota has an important impact on intestinal homeostasis and the regulation of the intestinal epithelial barrier integrity. Perturbations in the composition and function of gut microbiota have been associated with chronic diseases ranging from irritable bowel disease and metabolic disorders to neurological, cardiovascular and respiratory conditions (Tlaskalová‐Hogenová et al., 2011). New preventive and therapeutic approaches now include changing the composition of the gut microbiota with prebiotics, probiotics, postbiotics, reconstituting the bacterial population by faecal transplantation or adding antimicrobial agents to the diet (Shen et al., 2018).

The impact of probiotics is not limited to living bacteria but also includes the regulation of cellular homeostasis by microbial DNA, soluble proteins, cell wall components and metabolites (Adams, 2010; Aguilar‐Toalá et al., 2018). These microbe‐associated molecular patterns (MAMP) are recognised by the family of innate immune pattern recognition receptors (PRRs), such as Toll‐like receptors (TLRs) (Abreu, 2010). TLR signalling influences innate and adaptive immune regulation, which promotes pathogen clearance on the one hand and host–microbe symbiosis on the other (Spiljar, Merkler, & Trajkovski, 2017). However, TLRs are not only expressed in immune cells, such as antigen‐presenting cells (APCs), but also in intestinal epithelial cells (IEC; Price et al., 2018). On the apical surface of IECs, TLR2 recognises microbial membrane structures such as lipoteichoic acid, peptidoglycan and lipopeptides and is therefore involved in the detection of probiotics, which upon recognition trigger mitogen‐activated protein kinase (MAPK), nuclear factor kappa B (NF‐κB) and phosphatidylinositol 3‐kinase (PI3K) signalling cascades (Jiménez‐Dalmaroni, Gerswhin, & Adamopoulos, 2016; Maier, Anderson, Altermann, & Roy, 2018). TLR2 forms heterodimers with TLR1 and TLR6; however, its signalling cascades are also associated with TLR10 (Guan et al., 2010; Jin et al., 2007; Kang et al., 2009). According to recent studies, TLR10 is a negative regulator of TLR signalling and differs from other TLRs in the absence of a classical downstream signalling cascade leading to transcriptional activation of NF‐κB and proinflammatory cytokine production (Oosting et al., 2014). By suppressing the conventional MAPK signalling pathways, TLR10 thus plays an important role in immune tolerance (Jiang, Li, Hess, Guan, & Tapping, 2016). Because of its inhibitory effect on TLR1/TLR2 and TLR2/TLR6 signalling, TLR10 can regulate the immune response to a broad spectrum of microbial antigens.

Due to the difficulties in approving probiotic efficacy in clinical trials, current research is more focused on identifying probiotic mechanisms and host cell signalling pathways. Despite intensive research, the mechanisms behind cell signalling are not yet well understood. Our previous study on DSS‐induced colitic mouse model shows that a combination of two probiotic strains helped in the regeneration of the intestinal epithelium during pathogenesis via pathways that are known downstream targets of TLR2 (Paveljšek et al., 2018). In the present study, we aimed to reveal the TLR2‐mediated signalling pathways that are probably responsible for the regulation of the intestinal epithelial barrier. By using different in vitro setups of IECs challenged with probiotics, we investigated these signalling cascades triggered by four probiotic strains *Lactobacillus paragasseri* K7 isolated from the faeces of a 7‐day‐old infant (Treven, Trmčić, Matijašić, & Rogelj, 2014), *Limosilactobacillus fermentum* L930BB and *Bifidobacterium animalis* subsp. *animalis* IM386 isolated from the human intestinal mucosa (Čitar et al., 2015; Paveljšek et al., 2018), and *Lactiplantibacillus plantarum* WCFS1, human saliva isolate (Karczewski et al., 2010; Zheng et al., 2020). Our study confirmed former research findings that showed that activation of TLRs by commensal microorganisms is crucial for protection against intestinal damage (Bach, 2018). In addition, it also opened a new direction in mechanistic studies on the maintenance of intestinal homeostasis by probiotics.

## 2 RESULTS

### 2.1 Signalling of probiotic bacterial strains via TLR2‐NF‐κB

**FIGURE 1:**
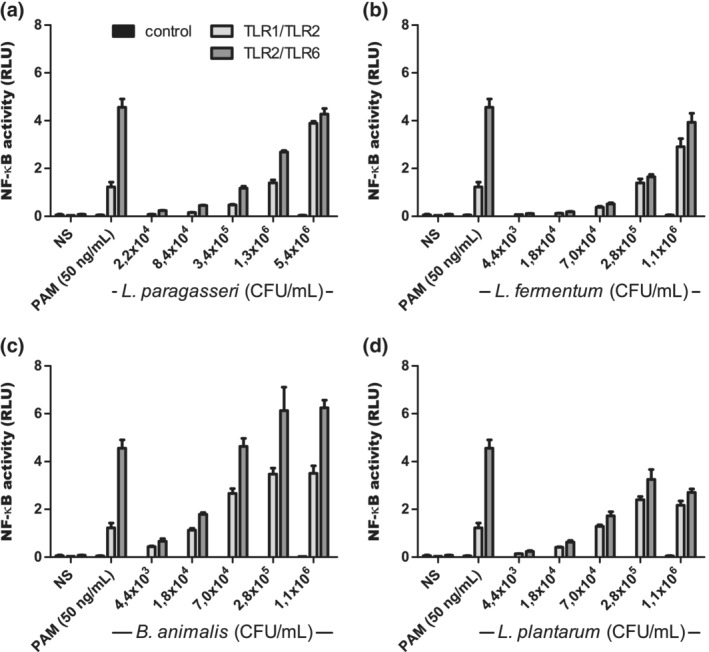
Activation of the transcription factor NF‐κB with selected probiotic strains through TLR2 heterodimers. The experiment was conducted in HEK293 cell line. *L. paragasseri* K7 (a), *L. fermentum* L930BB (b), *B. animalis* subsp. *animalis* IM386 (c) and *L. plantarum* WCFS1 (d). Mean values ± *SD* are shown. NS, non‐stimulated cells; RLU, relative luciferase units

**FIGURE 2:**
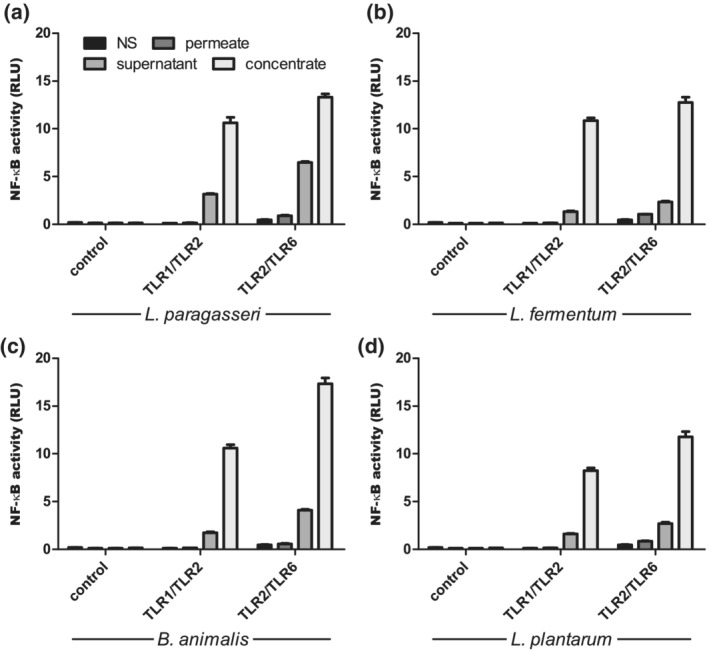
Activation of transcription factor NF‐κB with fractionated probiotic conditioned media through TLR2 heterodimers. Stimulation with supernatant, concentrated supernatant and permeate of growth medium after cultivation of probiotic strains *L. paragasseri* K7 (a), *L. fermentum* L930BB (b), *B. animalis* subsp. *animalis* IM386 (c) and *L. plantarum* WCFS1 (d) was performed in HEK293 cell line. Cell‐free supernatant was concentrated with 10 kDa cut‐off filter. Mean values ± *SD* are shown. NS, non‐stimulated cells; RLU, relative luciferase units

We used a dual luciferase reporter assay in the HEK293 cell line, transfected with TLRs and stimulated with the probiotic strains *L. paragasseri* K7 (K7), *L. fermentum* L930BB (L930BB), *B. animalis* subsp. *animalis* IM386 (IM386) and *L. plantarum* WCFS1 (WCFS1) at different concentrations. We found that all used probiotic strains activate the transcription factor NF‐κB through TLR1/TLR2 and through TLR2/TLR6 (Figure 1). IM386 triggered the highest activation of NF‐κB compared to other strains. The probiotic activation of NF‐κB through TLR2/TLR6 was higher than activation through TLR1/TLR2. Since TLR2 recognises lipoproteins, usually components of the bacterial surface, we were interested in whether the supernatant of bacterial growth medium and heat‐killed bacteria also trigger NF‐κB signalling cascades. We discovered that supernatant and heat‐killed bacteria trigger NF‐κB activation through TLR2 heterodimers in a similar concentration‐dependent manner as live bacteria. However, in the case of the heat‐killed strain WCFS1 and its supernatant, the activation of NF‐κB was lower compared to the activation with live bacteria (Figure S1). To determine the components that trigger the activation of NF‐κB, we further fractionated the supernatant with 10 kDa filters. We found that the components, responsible for TLR2‐induced NF‐κB activation, have a molecular weight of more than 10 kDa (Figure 2).

### 2.2 Probiotic inhibition of conventional TLR‐NF‐κB signalling pathway via TLR10 and stimulation of anti‐apoptotic pathways

**FIGURE 3:**
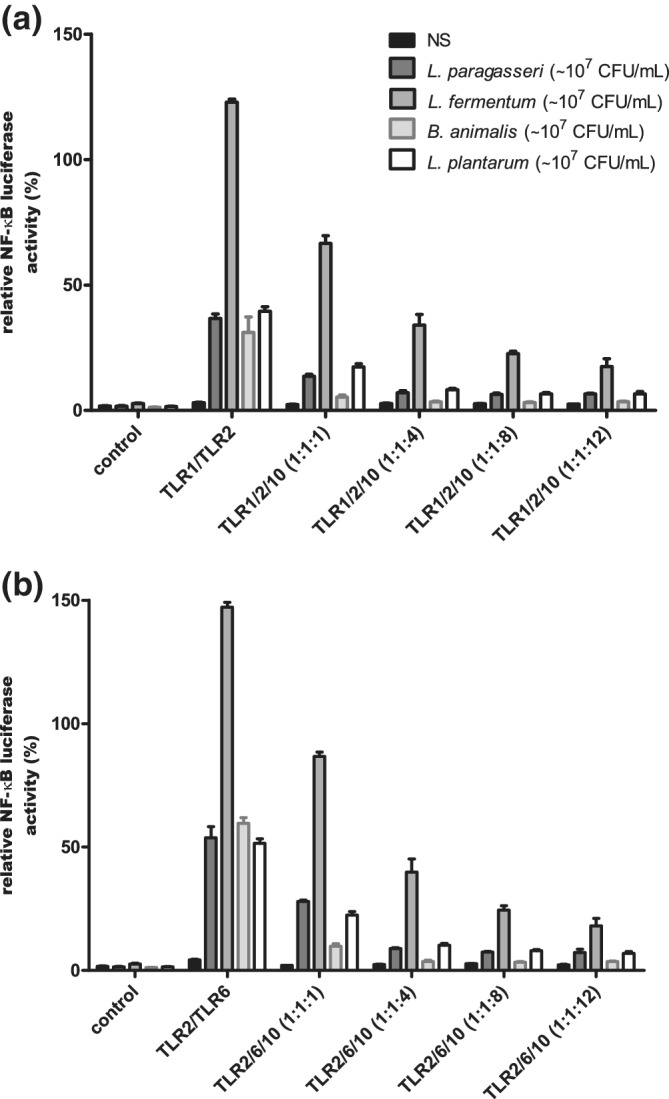
TLR10‐dependent reduction in activation of transcription factor NF‐κB after stimulation with probiotic strains. Receptor combinations TLR1/TLR2 (a) and TLR2/TLR6 (b) with different ratios of expressed TLR10 were used in HEK293 cell line. Mean values ± *SD* are shown. Used strains: *L. paragasseri* K7, *L. fermentum* L930BB, *B. animalis* subsp. *animalis* IM386, *L. plantarum* WCFS1. NS, non‐stimulated cells

**FIGURE 4:**
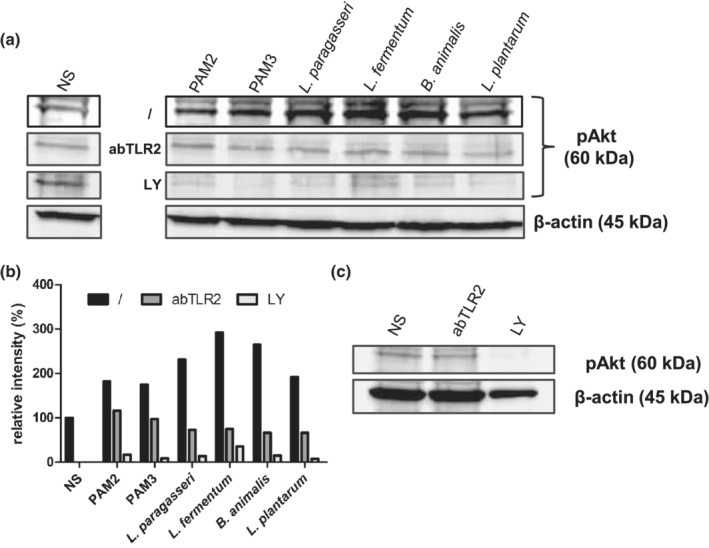
Impact of synthetic TLR2 ligands (PAM2, PAM3) and selected probiotic strains on phosphorylation of Akt (pAKT). The rate of Akt phosphorylation was observed after addition of TLR2 (abTLR2) and PI3K (LY) inhibitors in Caco‐2 cell line. Western blot for pAkt and β‐actin as internal control (a). Quantification of band intensity with software ImageJ (b). Western blot for pAkt in Caco‐2 cells, treated only with inhibitors (c). Used strains: *L. paragasseri* K7, *L. fermentum* L930BB, *B. animalis* subsp. *animalis* IM386, *L. plantarum* WCFS1. NS, non‐stimulated cells; /, no inhibition control

To test how probiotic strains affect TLR10 signalling cascades, we performed a dual luciferase reporter assay in the HEK293 cell line with expressed TLR1/TLR2, TLR2/TLR6 and increasing levels of expressed TLR10. We discovered that probiotic strains reduced the activation of NF‐κB in the presence of TLR10 via both TLR1/TLR2 and TLR2/TLR6 (Figure 3). To test the possible involvement of PI3K/Akt pathway in the inhibitory effect of TLR10, we used a non‐overexpression system on the IEC line Caco‐2. After addition of probiotic strains, phosphorylation of Akt, which is a measure of its activation by PI3K, was observed. In addition, the use of TLR2 and PI3K inhibitors reduced phosphorylation of Akt (Figure 4).

**FIGURE 5:**
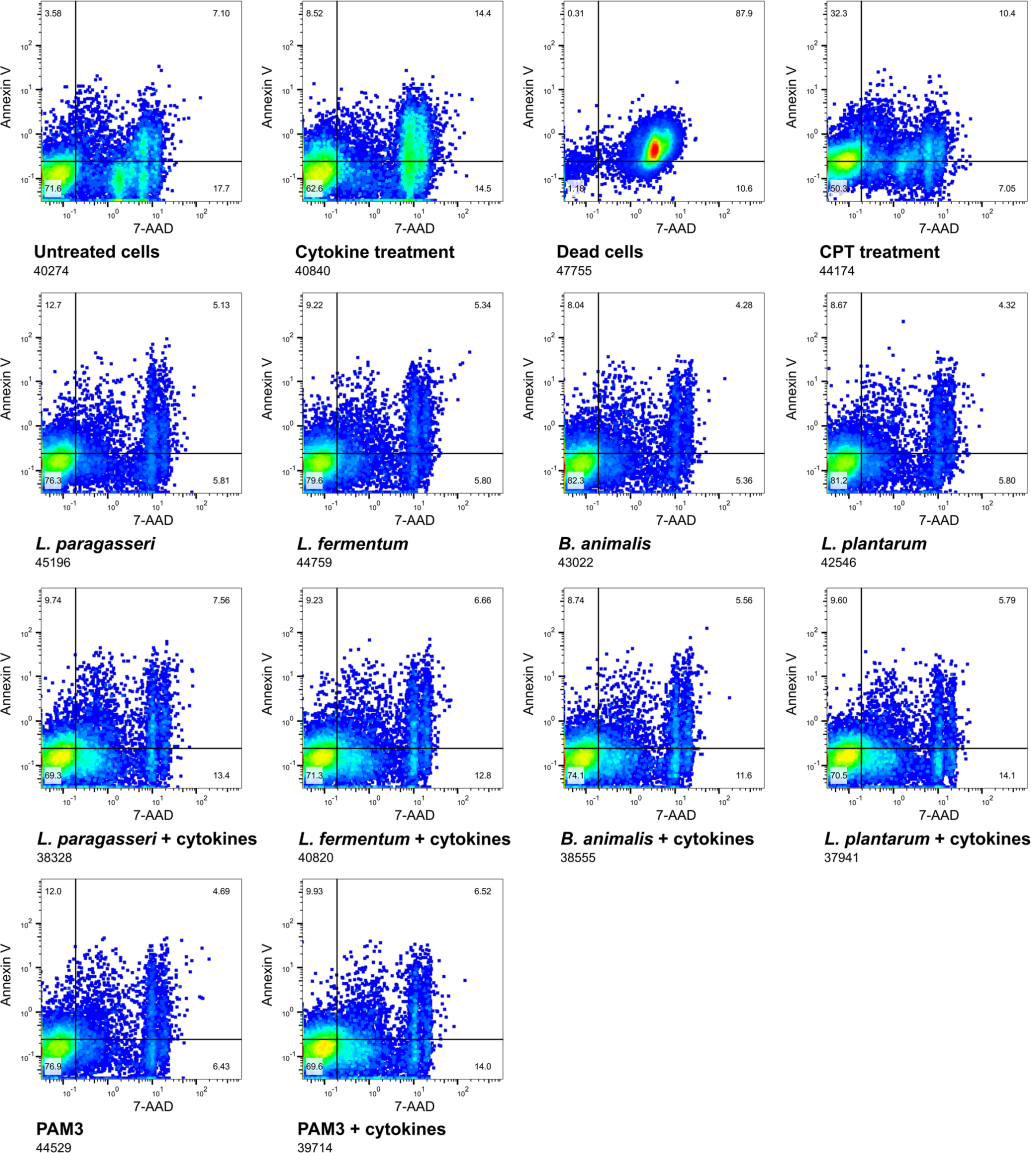
Impact of probiotic strains on viability of HT‐29 cells in stress conditions. Flow cytometry images of HT‐29 cell populations after different treatments. Apoptosis and necrosis were induced with addition of 100 ng/mL TNF‐α, 100 ng/mL IFN‐γ and 10 ng/mL IL‐1β. Used strains: *L. paragasseri* K7, *L. fermentum* L930BB, *B. animalis* subsp. *animalis* IM386, *L. plantarum* WCFS1. CPT, camptothecin

Since one of the most important signalling cascades via PI3K/Akt leads to the inhibition of apoptotic pathways, we tested whether the selected probiotic strains can influence cytokine‐induced apoptosis and necrosis. We used the IEC line HT‐29, which also expresses surface TLRs and shows a better inflammatory response to cytokines than the Caco‐2 cell line. The extent of apoptosis and necrosis was determined by analysing the live/dead HT‐29 cell populations with flow cytometry. The addition of probiotic strains to HT‐29 cell line reduced necrosis and late apoptosis caused by the addition of inflammatory cytokines (Figures 5 and S2). Furthermore, the TLR2 synthetic ligands and the probiotic strains had a similar protective effect on cell necrosis, proving TLR2‐mediated mechanism.

### 2.3 Probiotic impact on the intestinal epithelial barrier

**FIGURE 6:**
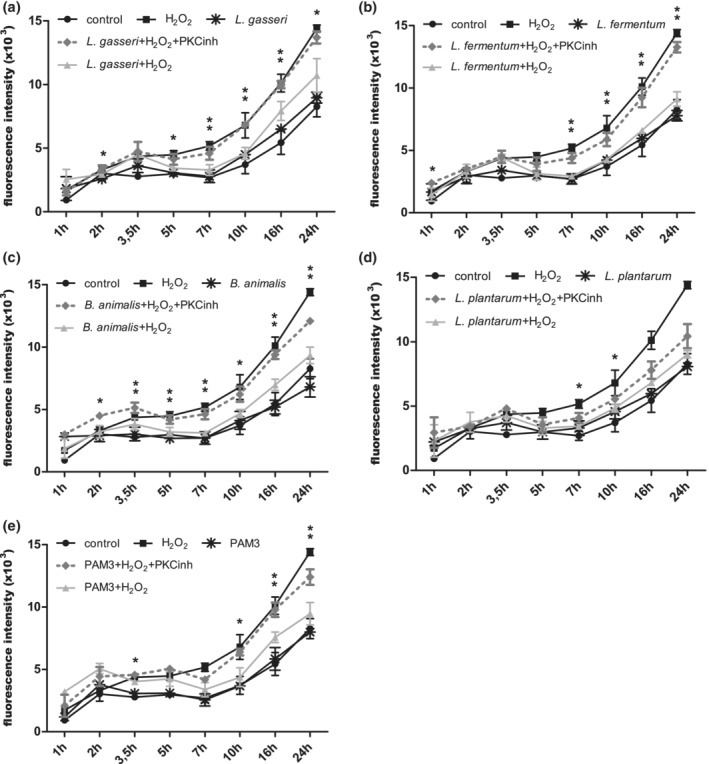
Effect of probiotics and TLR2 ligand on permeability of Caco‐2 cell monolayer after H_2_O_2_‐induced oxidative stress. *L. paragasseri* K7 (a), *L. fermentum* L930BB (b), *B. animalis* subsp. *animalis* IM386 (c), *L. plantarum* WCFS1 (d) and PAM3 (e). Mean values ± *SD* are shown. *p‐value ≤ .05 and **p‐value ≤ .01 between strain/PAM3 + H_2_O_2_ and strain/PAM3 + H_2_O_2_ + PKCinh. PKCinh, protein kinase C inhibitor Gӧ6983

We first investigated the possible protective effect of probiotic strains on the permeability of the H_2_O_2_‐disrupted Caco‐2 cell monolayer. We monitored the transition of FITC‐dextran from the apical to the basolateral compartment of semi‐permeable membranes. The addition of selected probiotic strains and the synthetic TLR2 ligand—Pam3Cys‐SK4 (PAM3) to the Caco‐2 cell line cultured on permeable supports did not alter the cell monolayer permeability (Figure 6). However, their addition to the H_2_O_2_‐disrupted Caco‐2 cells led to a reduction in permeability. Given that probiotics had the same protective effect as PAM3, we next investigated whether the probiotic influence on epithelial permeability is related to the PKC signalling pathway, a known TLR2‐dependent pathway that impacts on the paracellular sealing complex. We found that the addition of PKC inhibitor Gӧ6983 (PKCinh) reduced the protective effect of the probiotic strains, which verifies the relationship between probiotic activity and the PKC signalling pathway. In fact, permeability increased to the level of the cell monolayer exposed only to oxidative stress with H_2_O_2_ (Figure 6). However, the potency of inhibition was weaker for WCFS1.

**FIGURE 7:**
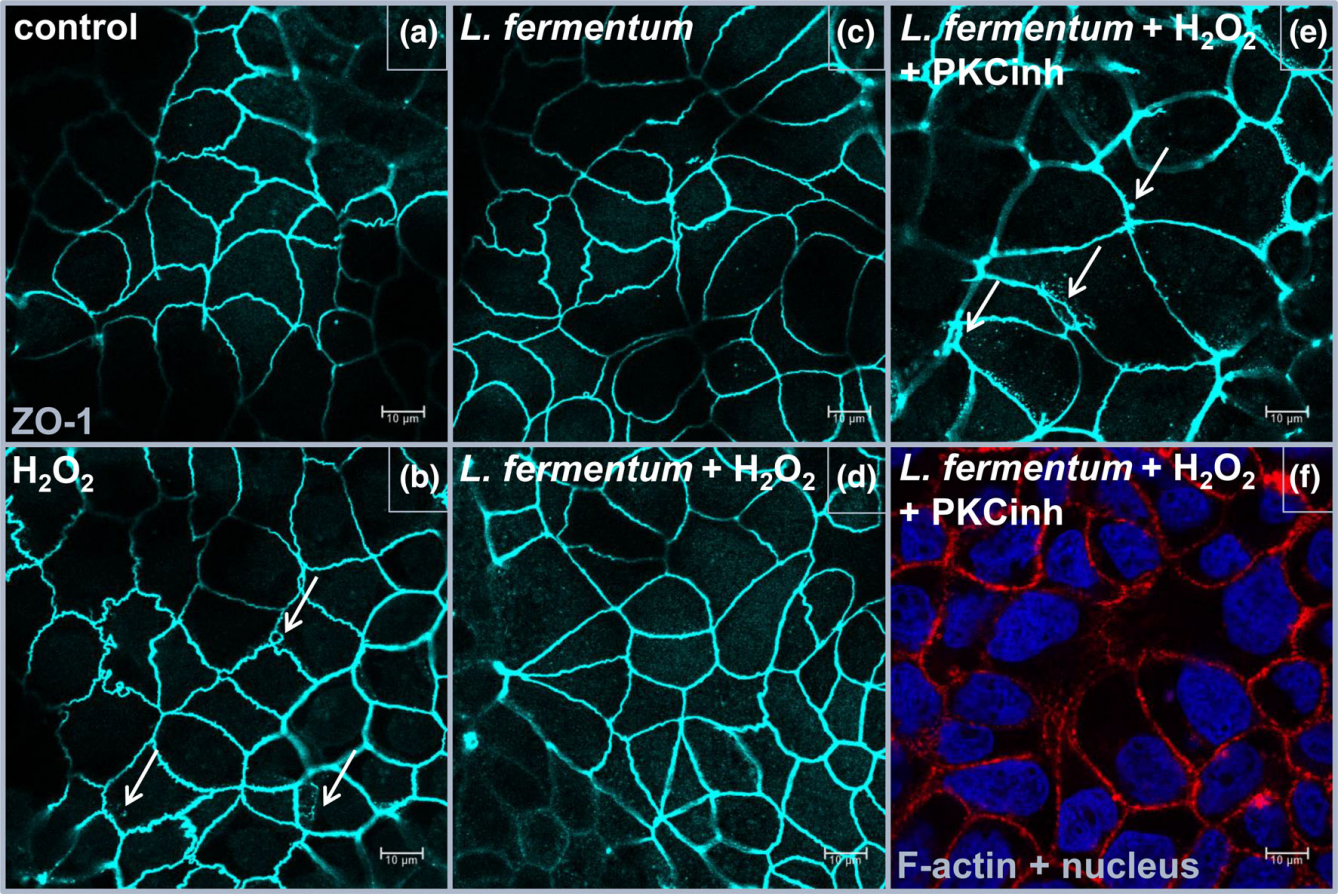
PKC‐dependent impact of *L. fermentum* L930BB strain on rearrangement of tight junction protein ZO‐1. Distribution of ZO‐1 (blue) in untreated and with H_2_O_2_ treated Caco‐2 cell line. Untreated cells—Control (a), cells treated with H_2_O_2_ (b), application of *L. fermentum* L930BB (c), application of *L. fermentum* L930BB and H_2_O_2_ (d), application of *L. fermentum* L930BB, H_2_O_2_ and protein kinase C inhibitor (e) stained also for nucleus and F‐Actin (f). Arrows indicate internalisation of ZO‐1. PKCinh, protein kinase C inhibitor Gӧ6983

Since TLR2 signalling cascades can influence the PKC‐mediated distribution of tight junctions (Figure S3), we investigated whether probiotic signalling through PKC is involved in this regulation as well. The localization of the fluorescently labelled cytoplasmic tight junction protein zonula occludens‐1 (ZO‐1) was examined by confocal microscopy in the Caco‐2 cell line. The addition of H_2_O_2_ caused the ZO‐1 protein to migrate away from the membrane site into the cell interior, where it no longer connects other transmembrane tight junctions with the actin cytoskeleton (Figure 7). This was observed as punctate staining in cytoplasm and as spacing/ring‐like staining pattern adjacent to the cell–cell interface. Irregular undulating patterns of ZO‐1 were also visible on the cell periphery. The preventive addition of the probiotic strains K7, L930BB, IM386 and WCFS1 reduced the H_2_O_2_‐induced intercellular spacing and internalisation of ZO‐1 and the addition of PKCinh prevented the protective effect of the probiotic strains (Figures 7, S4 and S5).

## 3 DISCUSSION

**FIGURE 8:**
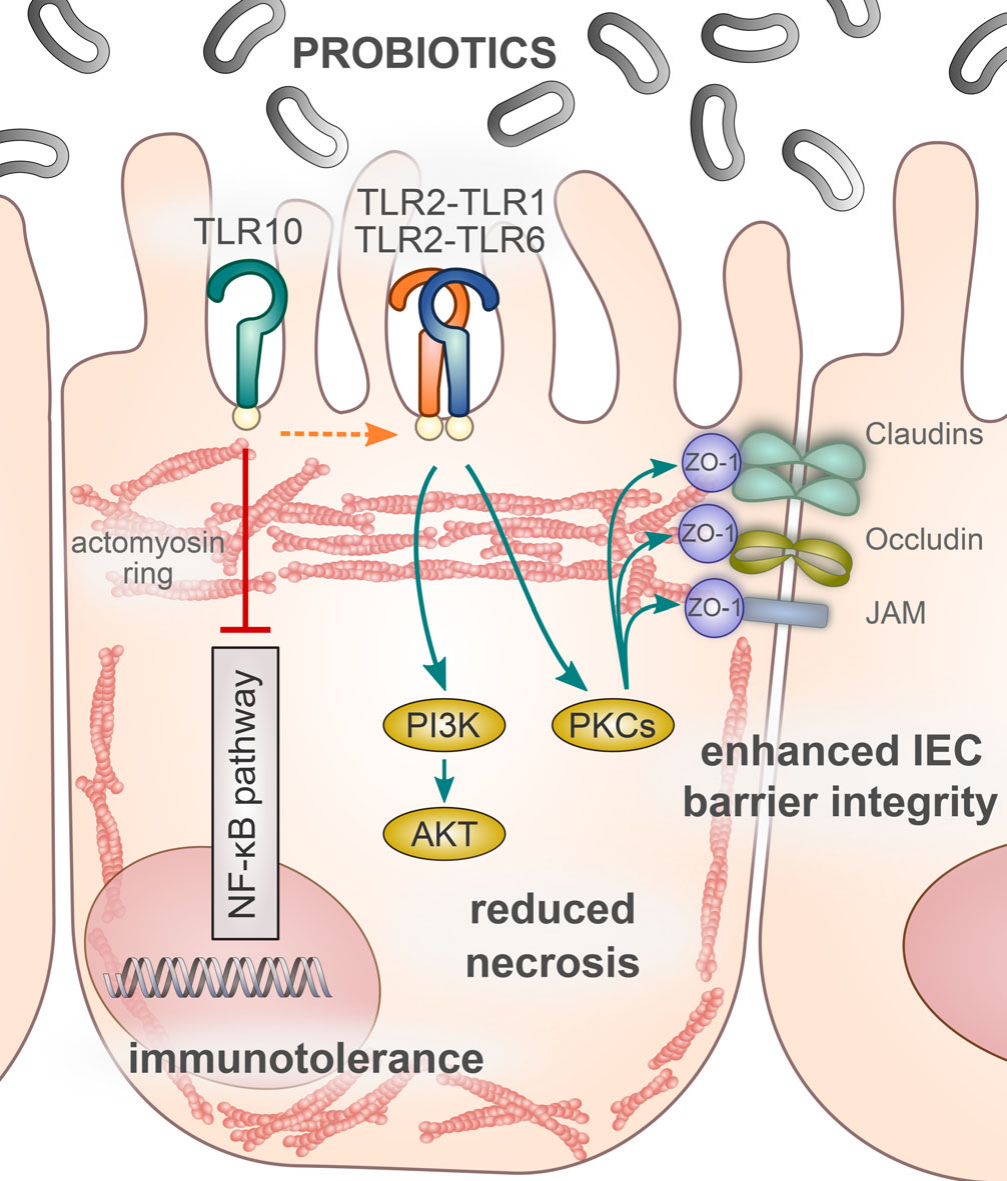
TLR2‐ and TLR10‐mediated probiotic signalling. TLR2‐dependent bacterial–cellular interactions presumably help to improve intestinal immune tolerance, reduce necrosis and enhance barrier integrity. Probiotic signalling through TLR10 probably blocks canonical NF‐κB cascades and promotes PI3/Akt pathway that reduces necrosis. Probiotics also mediate TLR2‐dependent PKC stabilisation of tight junctions and thus most likely contribute to the formation of the entire sealing complex and a stronger intestinal epithelial barrier. Akt, protein kinase B; IEC, intestinal epithelial cell; JAM, junction adhesion molecule; NF‐κB, nuclear factor kappa B; PI3K, phosphatidylinositol 3‐kinase; PKC, protein kinase C; TLR, Toll‐like receptor; ZO‐1, zonula occludens‐1

Beneficial effects of probiotic bacteria are often defined by the impact on the host gut microbiota, by the regulation of intestinal epithelial barrier function and the regulation of the immune response (Bermudez‐Brito, Plaza‐Díaz, Muñoz‐Quezada, Gómez‐Llorente, & Gil, 2012). Cell lines as model systems are used in a variety of research studies dealing with the protective effects of probiotics, ranging from studies of cell signal transduction to the administration of drugs and processes of intestinal diseases. Although they only approximate the properties of in vivo cells in tissues (Engle, Goetz, & Alpers, *1998*), these models also offer advantages, including more controlled conditions and robustness. However, for more thorough conclusions, it is still necessary to start from more complex models that better reflect the native state. Therefore, we have based our study on the mouse experiment from our previous publication. In a previous study, we performed a global gene expression analysis in a DSS‐induced mouse model of colitis and emphasised that the beneficial probiotic effect was probably associated with TLR2 downstream signalization (Paveljšek et al., 2018). Now, we mainly focused on the probiotic modulation of innate immune signalling and the intestinal epithelial barrier maintenance. We investigated the protective effect of probiotics in inflammatory conditions by determining cell viability and cell monolayer permeability and observing the distribution of tight junction protein ZO‐1 and actin cytoskeleton. We discovered that the selected bacterial strains have a similar effect on the potential TLR2 signalling at the intestinal epithelial interface and elicit three probiotic properties: (a) general tolerance and immune regulation via TLR10, (b) cell survival and reduced necrosis via PI3K/Akt pathway and (c) improved barrier integrity via PKC and ZO pathway (Figure 8).

Besides the poorly understood biological function of TLR10, which in contrast to other TLRs mainly has an inhibitory effect, there are also no known ligands for this receptor (Jiang et al., 2016). We showed that the co‐expression of TLR10 with TLR1/TLR2 as well as with TLR2/TLR6 had an inhibitory effect on NF‐κB‐mediated response to probiotics, which has not been shown in the literature before. The signalling of probiotic strains through TLR10 may therefore be considered as an additional immune‐regulatory effect that promotes remission in autoimmune intestinal diseases. In addition, the TLR2 signalling cascades of live bacteria were similar to those of the heat‐killed and cell‐free supernatant. Showing, that the metabolites and/or cell wall components released by probiotics, defined by the term postbiotics, represent an important component of innate immune regulation. These signalling cascades, possibly triggered by the TLR2‐TLR10 assembly, include the PI3K/Akt anti‐apoptotic pathway that inhibits MAPKs/NF‐κB signal transduction, thereby providing general tolerance to commensals on the mucosal barrier.

In IECs, the absence of TLR2 causes inadequate PI3K/Akt signalling and disruption of the intracellular anchoring complex. This leads to increased apoptosis, detachment of colonocytes and consequently to a greater permeability of the epithelial barrier, resulting in mucosal inflammation (Cario, Gerken, & Podolsky, 2007). Indeed, we showed that probiotics induce signalling through PI3K/Akt and reduce necrosis in inflammatory conditions and thereby help to maintain the barrier integrity.

We also examined the probiotic influence in stress conditions on activation of PKC—another TLR2‐mediated downstream signalling pathway that can prevent internalisation or translocation of the cytosolic tight junction protein ZO‐1 from the plasma membrane and is also important for the epithelial barrier integrity (Corr et al., 2014; Gu et al., 2016). IECs express a number of PKCs that regulate the phosphorylation state and localization of several tight junction proteins (Zyrek et al., 2007). According to Cario, Gerken, and Podolsky (2004), stimulation of the TLR2 pathway leads to activation of PKCα and PKCδ, and subsequently to redistribution of ZO‐1 and improvement of cell monolayer integrity. In our study, both probiotic strains and TLR2 ligands triggered the activation of specific PKC isoforms, which had an influence on the localization of ZO‐1 and consequently on the reduction of cell monolayer permeability, suggesting and confirming that TLR2 signalization is responsible for this downstream effect.

Interestingly, the same mechanism of action and the same TLR2 signalling cascades were observed for all selected probiotics. This can be explained by the fact that the MAMPs that trigger signalization have a similar basic structure and are conserved among a larger group of probiotics (Sanders, Benson, Lebeer, Merenstein, & Klaenhammer, 2018). It is also important to keep in mind that the bacterial cell surface is a dynamic property and the expression of bacterial surface components may vary due to the host environment (Lebeer, Vanderleyden, & De Keersmaecker, 2010). Furthermore, recent research demonstrates that some of the molecular complexes responsible for immuno‐modulation in host cells may be common to a larger taxonomic group (Lebeer et al., 2018), which additionally contributes to the shared probiotic properties, also considering that TLR2 recognises a broad spectrum of ligands (Jiménez‐Dalmaroni et al., 2016). Besides, selected probiotic strains also showed no NF‐κB activity via TLR5 and TLR9 (Figure S6), thus excluding other TLR signalling, since lactic acid bacteria and bifidobacteria do not possess LPS (TLR4 signalization) and the remaining TLRs are associated mainly with viral infections. Certainly, some important mechanisms are only present in a few probiotic strains, but some molecules are not strain‐specific and have homologues among different strains or even other species. For example, the secretory proteins p40 and p75 derived from *Lacticaseibacillus rhamnosus* GG have homologues in the genomes of *L. casei*, *L. paracasei* and *L. rhamnosus* (Bäuerl, Pérez‐Martínez, Yan, Polk, & Monedero, 2011). However, the fact that the heat‐killed WCFS1 did not elicit the same NF‐κB activity as live culture, brings up the question whether the ligands for TLR2 of WCFS1 are structurally different and heat sensitive compared to the other three probiotic strains.

In addition to the nature of ligand, the activation of specific signalling pathways and the outcome of MAMP–PRR interactions also depend on the type of host cell and the use of different cell lines (Gagnon, Zihler Berner, Chervet, Chassard, & Lacroix, 2013). Variances in the downstream signalling cascades occur when we compare the response of immune cells and of IECs, which are polarised and have lower expression of surface PRRs (Price et al., 2018). For example, TLR2 signalling via the MyD88 pathway in IECs usually has a protective effect by promoting immune tolerance to microbiota. In contrast, the TLR signalling of immune cells has an inflammatory response to bacterial ligands, which helps to prevent bacterial invasion through the epithelial monolayer (Abreu, 2010; Goto, 2019; Yu & Gao, 2015).

We would also like to point out the limitations of this type of studies, where it is sometimes difficult to draw conclusions with experiments on cell models only, as these do not cover the effects on the overall regulation of the immune system. It is also difficult to compare the results of different studies where different protocols as well as different cell lines are used. Furthermore, cell lines only represent an approximation of the real biological state, so it is essential that these studies are supported by more complex models such as organoids or experiments on animal models, which were also the starting point of our study (Paveljšek et al., 2018).

In conclusion, we were able to show that the strains L930BB, IM386, K7 and WCFS1 induce the TLR2 signalling, activate anti‐apoptotic pathways, impact on tight junction proteins and the actin cytoskeleton and thus contribute to the regeneration of the intestinal epithelium and the modulation of the mucosal barrier of the immune system. The results of our study confirmed and connected important observations from other studies and are a step forward in understanding probiotic communication with host cells and its beneficial effects.

## 4 EXPERIMENTAL PROCEDURES

### 4.1 Cell lines and bacterial strains

Human embryonic kidney cell line HEK293 (ATCC CRL‐1573), human colon adenocarcinoma cell line Caco‐2 (ATCC HTB‐37) and human colon adenocarcinoma cell line HT‐29 (ATCC HTB‐38) were cultured in complete media [Dulbecco's Modified Eagle Medium (for HEK293 and Caco‐2) or McCoy 5a (for HT‐29), 10% heat‐inactivated foetal bovine serum (FBS, Gibco, Invitrogen)] in 5% CO_2_ at 37°C. Media for HT‐29 and Caco‐2 cell lines also included 1% penicillin–streptomycin (Thermo Fisher Scientific) which was excluded from the media 2 days prior to the experiments. Cell viability was determined prior plate seeding by counting trypan blue‐stained samples using the Countess instrument (Thermo Fisher Scientific). The survival values were above 90% in all experiments.

*Limosilactobacillus fermentum* L930BB (DSMZ 26139) and *B. animalis* subsp. *animalis* IM386 (DSMZ 26137) strains were obtained from bioptic samples of colonic mucosa and deposited in a culture collection at the Biotechnical faculty, University of Ljubljana and in the Leibniz‐Institut—DSMZ. *Lactobacillus paragasseri* K7 strain was obtained from infant faeces and deposited in a culture collection at the Biotechnical faculty, University of Ljubljana and in the Czech Collection of Microorganisms under accession number CCM7710. *Lactiplantibacillus plantarum* WCFS1 strain is deposited in the Laboratorium voor Microbiologie, Universiteit Gent under accession number LMG9211. All bacterial strains were cultured at 37°C in De Man, Rogosa, and Sharpe (MRS) broth (Merck). For the stimulation experiments, overnight bacterial cultures were centrifuged (10 min, 8000 rcf), pellet was washed with PBS, centrifuged and resuspended in DMEM with 10% FBS. After described preparation, bacterial cell concentration was adjusted by measuring the optical density at 650 nm. When cell‐free supernatant was used for stimulation of HEK293 cell line, bacterial strains were cultured in DMEM. Cell‐free supernatant was prepared with centrifugation of overnight culture (10 min, 8000 rcf) and sterile filtration through 0.2 μm filter (Merck). In additional experiments, cell‐free supernatant was concentrated with 10 kDa cut‐off filter device Amicon (Merck) for 20 min at 3850 rcf. Heat‐killed bacterial cells were treated for 30 min at 80°C.

### 4.2 Dual luciferase reporter assay

HEK293 cells were seeded in 96‐well plates (Corning) at 2.7 × 10^4^ cells per well. Cells reached 50% confluence overnight and were at that point transiently transfected with different combinations of pUNO plasmids expressing TLR1, TLR2, TLR6 (5 ng DNA per well), TLR10 (5–60 ng DNA per well) or pcDNA3 (Invivogen), ELAM1‐luciferase reporter plasmid (50 ng DNA per well) (Kirschning, University of Duisburg‐Essen, Germany), and phRL‐TK (5 ng DNA per well, Promega) using the jetPEI transfection reagent (Polyplus Transfection). The plasmids pUNO‐hTLR5 (10 ng DNA per well) to verify signalling via TLR5 and pUNO‐hTLR9 (20 ng DNA per well) with pUNO1‐hUNC93B1 (5 ng DNA per well) to verify signalling via TLR9 were used by the same transfection protocol. After 24 hr, the culture medium was replaced with fresh medium, and the cells were stimulated with probiotic strains or TLR2 agonists (InvivoGen), Pam3Cys‐SK4 (PAM3) (50 ng/mL) or Pam2Cys‐SK4 (PAM2) (5 ng/mL), for 18 hr. In case of TLR5 signalization the ligand *S. typhimurium* flagellin (SaTy) (50 ng/mL) was used and in case of TLR9 signalization the ligand CpG‐ODN 2006 Biotin (10 μM) was used. The cells were lysed in passive lysis buffer (Promega) and analysed for reporter gene activities using a dual luciferase reporter assay. The Renilla luciferase reporter (phRL‐TK) was used as an internal control for normalisation. Standard deviation was calculated from four replicates. The experiment was performed in three biological replicates.

### 4.3 Western blot

Caco‐2 cells (24th–26th passage) were seeded in a 6‐well plate (Corning) at a density of 3.3 × 10^5^ cells per well and cultured for 10 days until complete confluency with media changes every other day. Two days before the experiment antibiotic‐free media with 200 μM LY294002 (LY, Invivogen) or 5 μg/mL PAb hTLR2 (abTLR2, Invivogen) was added to the designated test wells. One hour before the experiment, the media with inhibitors was changed again. Cells were stimulated for 1 hr with probiotic strains (10^7^ CFU per well) or TLR2 ligands, PAM2 (2 μg/mL) or PAM3 (20 μg/mL). After stimulation, the cells were washed twice with cold PBS and lysed on ice for 30 min in 170 μL of RIPA buffer (Thermo Fisher Scientific) containing 1:100 protease inhibitors (Thermo Fisher Scientific) and 1:5 phosphatase inhibitors (Merck Millipore). Cell debris was removed by centrifugation at 13400 rpm, 4°C for 30 min. The total protein concentration in the supernatant was determined using the BCA assay. Further, proteins from the supernatant were separated by SDS‐PAGE and transferred to a Hybond ECL nitrocellulose membrane (GE Healthcare). The membrane was incubated in blocking buffer [1 × PBS, 0.1% Tween 20, 0.2% I‐Block (Tropix)] overnight at 4°C. The membranes were incubated with primary antibodies diluted in blocking buffer for 90 min, washed (1× PBS, 0.1% Tween 20), and incubated with secondary antibodies for 45 min at room temperature. Secondary antibodies were detected with the ECL Western blotting detection reagent (GE Healthcare), according to the manufacturer's protocol. The primary antibodies were rabbit Phospho‐Akt Thr308 (pAkt) (9275S, Cell Signaling Technology) and mouse β‐aktin 8H10D10 (3700, Cell Signaling Technology) diluted 1:1000. Secondary antibodies were HRP‐conjugated goat anti‐rabbit IgG (ab6721, Abcam) and HRP‐conjugated goat anti‐mouse IgG (sc‐2005, Santa Cruz) diluted 1:5000. The experiment was performed in three biological replicates.

### 4.4 Flow cytometry

HT‐29 cells (16th passage) were 5seeded in a 24‐well plate (Corning) at a density of 5 × 10^5^ cells per well. Media was changed daily, and cells were cultured for 3–4 days under standard culture conditions until complete confluency. Cells were stimulated for 1 hr with probiotic strains (10^7^ CFU per well) or PAM3 (2 μg/mL). After stimulation, the media was removed and cells were treated for 5 hr with cytokines (100 ng/mL TNF‐α, 100 ng/mL IFN‐γ, 10 ng/mL IL‐1β). Control for apoptosis were camptothecin‐treated cells (CPT, 60 μM) and control for necrosis were heat‐treated cells (20 min, 60°C). After the treatment, the cells were trypsinised and media was removed. The cells were stained according to the manufacturer's instructions with Annexin V Apoptosis Detection Kit eFluor 450 (eBioscience) and analysed within 4 hr on flow cytometer (CyFlow space, Partec). Data was analysed with FlowJo software (Tree Star). The experiment was performed in two biological replicates.

### 4.5 Fluorometric analysis of cell monolayer permeability

Caco‐2 cells (26th passage) were seeded on 12‐well semi‐permeable membranes (Transwell, Corning) at a density of 7 × 10^4^ cells per well and cultured for 21 days until differentiation with media changes every other day. The designated group of cells was treated with PKC inhibitor Gӧ6983 (PKCinh) (5 μg/mL, Sigma) 1 hr before the experiment. Afterwards, the probiotic strains (10^7^ CFU per well) or PAM3 (20 μg/mL) were added to the apical compartment and incubated for 1 hr. After incubation H_2_O_2_ (750 μM) was added to both compartments and 3 kDa FITC‐dextran (0.2 mg/mL, Thermo Fisher Scientific) was added to the apical compartment. Samples were taken from the basolateral compartment and the fluorescence intensity was measured on a Synergy MX microtiter plate reader (Tecan). The experiment was performed in two biological replicates. Statistical analysis was carried out using unpaired Student's *t* test. A *p*‐value of ≤.05 and ≤.01 was considered statistically significant.

### 4.6 Confocal microscopy

Caco‐2 cells were seeded onto eight‐well tissue culture chambers (Ibidi) at a density of 1.78 × 10^4^ cells per well and cultured for 21 days with media changes every other day. The designated group of cells was treated with PKCinh (20 μM, Sigma) 1 hr before the experiment. Subsequently, the cells were stimulated for 1 hr with probiotic strains (10^7^ CFU per well) or PAM3 (20 μg/mL). After the stimulation, the media was removed, and the cells were washed with PBS. Fresh media with 100 μM H_2_O_2_ was added for 2 hr. Cells were washed again with PBS, fixed (4% paraformaldehyde) and permeabilised (0.1% Triton‐x100, Sigma). The cells were stained for F‐actin, ZO‐1 and nucleus with Phalloidin Alexa Fluor 647 (8940, Cell Signaling Technology), ZO‐1 Alexa Fluor 488 (ZO1‐1A12, Thermo Fisher Scientific) and DAPI (D21490, Thermo Fisher Scientific). Images were acquired using the Leica TCS SP5 confocal microscope equipped with an HCX Plan‐Apochromat lambda blue ×63 oil‐immersion objective with NA 1.4 (Leica Microsystems). The images were processed with LAS AF software (Leica Microsystems). The experiment was performed in three biological replicates.

## CONFLICT OF INTEREST

The authors declare no conflicts of interest.

## AUTHOR CONTRIBUTIONS

Diana Paveljšek, Mojca Benčina and Karolina Ivičak‐Kocjan performed the experiments and analysed the data. Roman Jerala and Irena Rogelj conceived the experiments. Diana Paveljšek and Primož Treven wrote the manuscript with input from the other authors.

## Supporting information


**Figure S1**. Probiotic activation of NF‐κB through different TLR1, TLR2, TLR6 and TLR10 receptor combinations. Stimulation with live and heat‐killed bacterial cells and with supernatant of growth medium after cultivation of probiotic bacteria *L. paragasseri* K7 (A), *L. fermentum* L930BB (B), *B. animalis* subsp. *animalis* IM386 (C) and *L. plantarum* WCFS1 (D) was used in HEK293 cells. Mean values ± standard deviation are shown.
**Figure S2**. Graphical display of the percentage of live, apoptotic and necrotic HT‐29 cells at different treatments. Data from flow cytometry analysis. Apoptosis and necrosis were induced with addition of 100 ng/mL TNF‐α, 100 ng/mL IFN‐γ and 10 ng/mL IL‐1β. Controls (A), synthetic TLR2 ligand – PAM3 (B), *L. paragasseri* K7 (C), *L. fermentum* L930BB (D), *B. animalis* subsp. *animalis* IM386 (E) and *L. plantarum* WCFS1 (F). NS – non‐stimulated cells, CPT – cells treated with camptothecin, HT – heat treated cells.
**Figure S3**. PKC‐dependent impact of synthetic TLR2 ligand – PAM3 on rearrangement of ZO‐1 and actin. Distribution of ZO‐1 (blue) and F‐actin (red) in untreated and with H_2_O_2_ treated Caco‐2 cell line after stimulation with PAM3. Arrows indicate internalization of ZO‐1 and interruptions in the actin ring. PKCinh – protein kinase C inhibitor Gӧ6983.
**Figure S4**. PKC‐dependent impact of selected probiotic strains on rearrangement of tight junction protein ZO‐1 (blue). Distribution of ZO‐1 in untreated and with H_2_O_2_ treated Caco‐2 cell line after probiotic stimulation. Arrows indicate internalization of ZO‐1. Presented strains: *L. paragasseri* K7, *B. animalis* subsp. *animalis* IM386, *L. plantarum* WCFS1. PKCinh – protein kinase C inhibitor Gӧ6983.
**Figure S5**. PKC‐dependent impact of selected probiotic strains on rearrangement of F‐actin. Distribution of F‐actin (red) in untreated and with H_2_O_2_ treated Caco‐2 cell line after probiotic stimulation, stained also for nucleus. Arrows indicate interruptions in the actin ring or dead cells. Used strains: *L. paragasseri* K7, *L. fermentum* L930BB, *B. animalis* subsp. *animalis* IM386, *L. plantarum* WCFS1. PKCinh – protein kinase C inhibitor Gӧ6983.
**Figure S6**. Evaluation of the transcription factor NF‐κB activity after stimulation with selected probiotic strains through TLR5 and TLR9. TLR5 stimulation (A), TLR9 stimulation (B). The experiment was conducted in HEK293 cell line. Used strains: *L. paragasseri* K7, *L. fermentum* L930BB, *B. animalis* subsp. *animalis* IM386, *L. plantarum* WCFS1. Mean values ± standard deviation are shown. NS – non‐stimulated cells, ODN – CpG‐ODN 2006 Biotin, RLU – relative luciferase units, SaTy – *S. typhimurium* flagellin.

## Data Availability

Additional data available on request from the authors.
