# Supplementary material for: Distinctive probiotic features share common TLR2‐dependent signalling in intestinal epithelial cells
Source: Cell Microbiol. 2020 Oct 1;23(1):e13264. doi: 10.1111/cmi.13264 (PMC7757178; doi:10.1111/cmi.13264)
Supplement: Supplementary file 1 — Figure S1. Probiotic activation of NF‐κB through different TLR1, TLR2, TLR6 and TLR10 receptor combinations. Stimulation with live and heat‐killed bacterial cells and with supernatant of growth medium after cultivation of probiotic bacteria L. paragasseri K7 (A), L. fermentum L930BB (B), B. animalis subsp. animalis IM386 (C) and L. plantarum WCFS1 (D) was used in HEK293 cells. Mean values ± standard deviation are shown. Figure S2. Graphical display of the percentage of live, apoptotic and necrotic HT‐29 cells at different treatments. Data from flow cytometry analysis. Apoptosis and necrosis were induced with addition of 100 ng/mL TNF‐α, 100 ng/mL IFN‐γ and 10 ng/mL IL‐1β. Controls (A), synthetic TLR2 ligand – PAM3 (B), L. paragasseri K7 (C), L. fermentum L930BB (D), B. animalis subsp. animalis IM386 (E) and L. plantarum WCFS1 (F). NS – non‐stimulated cells, CPT – cells treated with camptothecin, HT – heat treated cells. Figure S3. PKC‐dependent impact of synthetic TLR2 ligand – PAM3 on rearrangement of ZO‐1 and actin. Distribution of ZO‐1 (blue) and F‐actin (red) in untreated and with H2O2 treated Caco‐2 cell line after stimulation with PAM3. Arrows indicate internalization of ZO‐1 and interruptions in the actin ring. PKCinh – protein kinase C inhibitor Gӧ6983. Figure S4. PKC‐dependent impact of selected probiotic strains on rearrangement of tight junction protein ZO‐1 (blue). Distribution of ZO‐1 in untreated and with H2O2 treated Caco‐2 cell line after probiotic stimulation. Arrows indicate internalization of ZO‐1. Presented strains: L. paragasseri K7, B. animalis subsp. animalis IM386, L. plantarum WCFS1. PKCinh – protein kinase C inhibitor Gӧ6983. Figure S5. PKC‐dependent impact of selected probiotic strains on rearrangement of F‐actin. Distribution of F‐actin (red) in untreated and with H2O2 treated Caco‐2 cell line after probiotic stimulation, stained also for nucleus. Arrows indicate interruptions in the actin ring or dead cells. Used strains: L. paragasseri [file CMI-23-e13264-s001.pdf]

Distinctive probiotic features share common TLR2-dependent signalling in intestinal epithelial cells

Supporting Figures

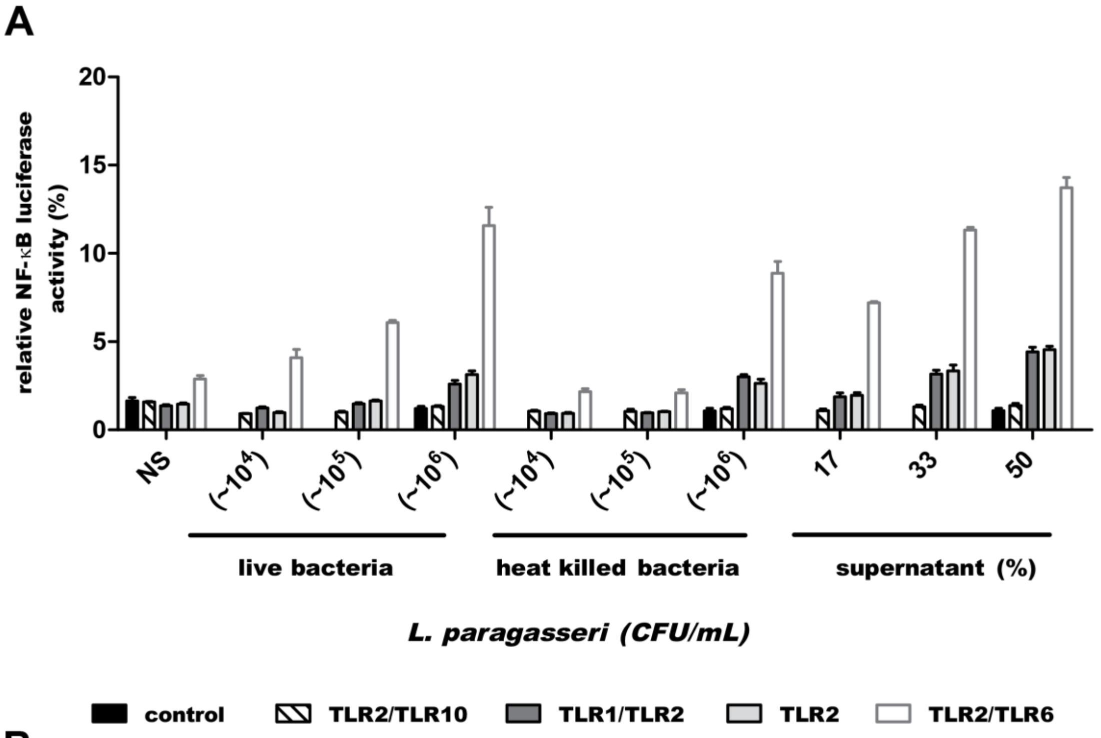

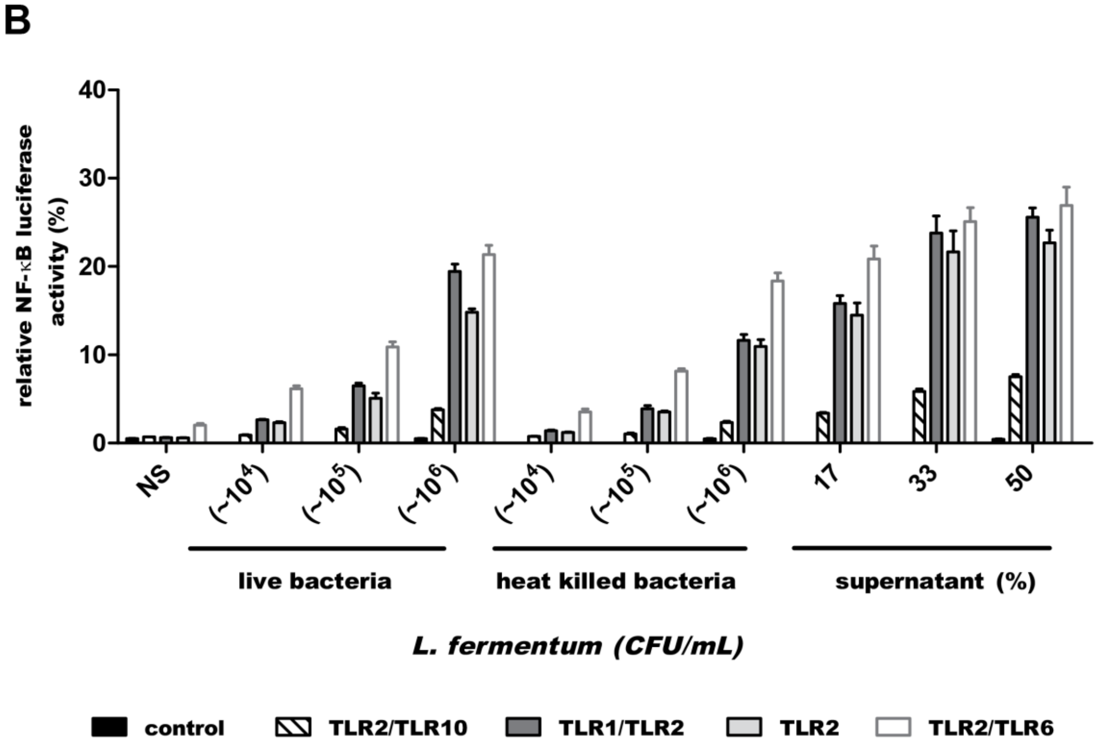

C

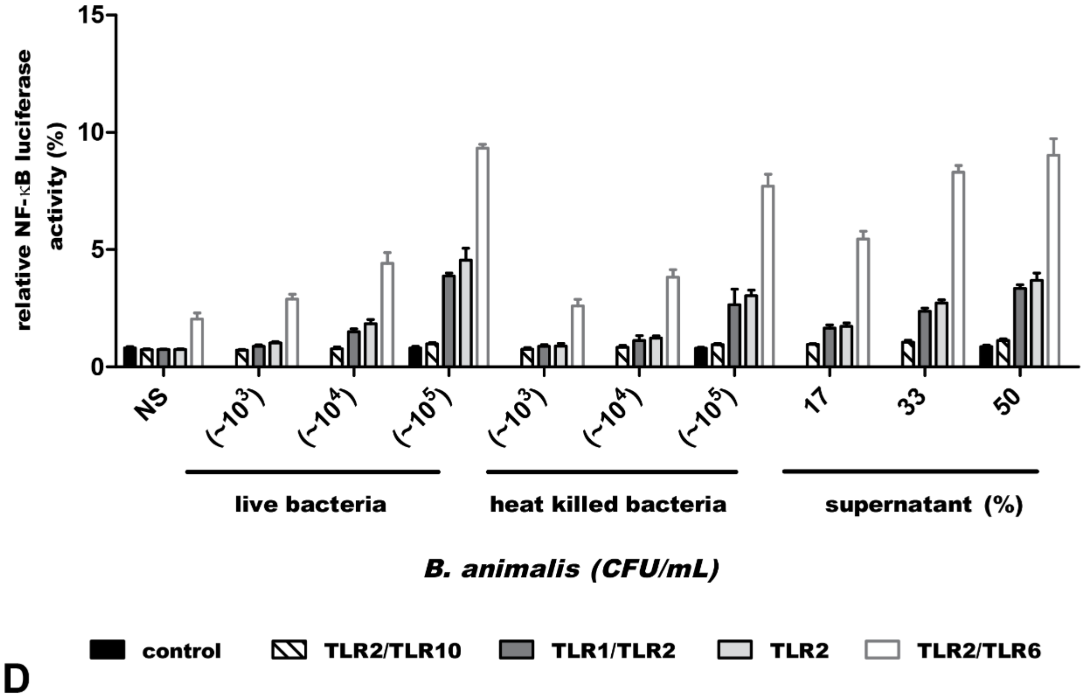

D

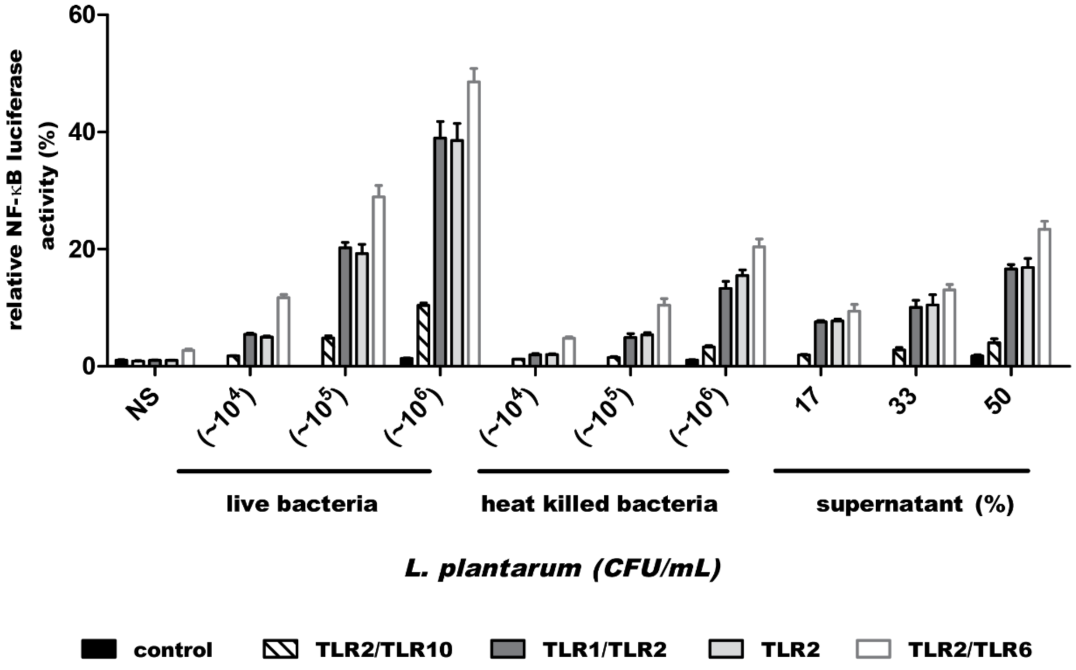

Figure S1: Probiotic activation of NF-κB through different TLR1, TLR2, TLR6 and TLR10 receptor combinations. Stimulation with live and heat-killed bacterial cells and with supernatant of growth medium after cultivation of probiotic bacteria *L. paragasseri* K7 (A), *L. fermentum* L930BB (B), *B. animalis* subsp. *animalis* IM386 (C) and *L. plantarum* WCFS1 (D) was used in HEK293 cells. Mean values ± standard deviation are shown.

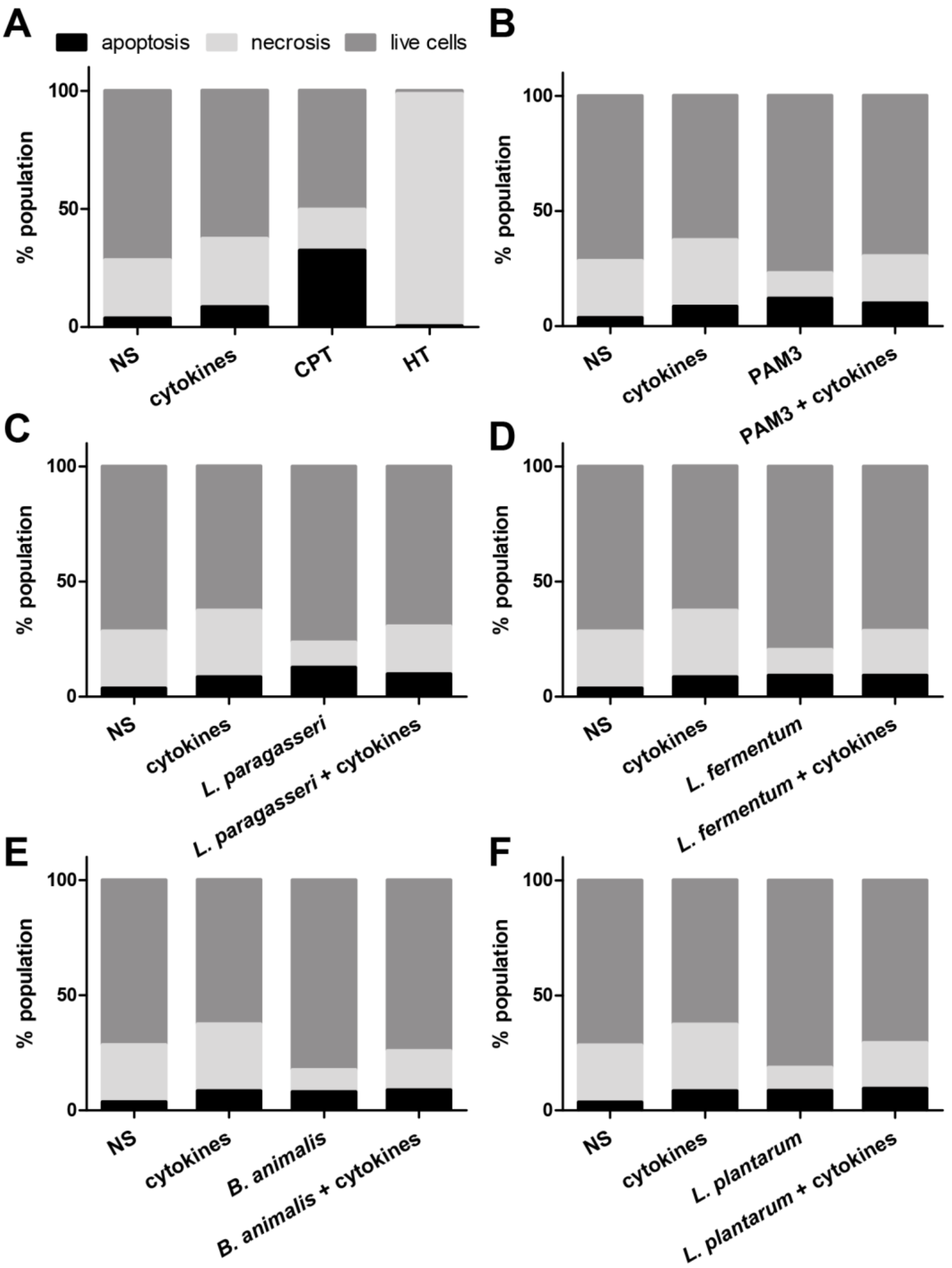

Figure S2: Graphical display of the percentage of live, apoptotic and necrotic HT-29 cells at different treatments. Data from flow cytometry analysis. Apoptosis and necrosis were induced with addition of 100 ng/mL TNF-α, 100 ng/mL IFN-γ and 10 ng/mL IL-1β. Controls (A), synthetic TLR2 ligand – PAM3 (B), *L. paragasseri* K7 (C), *L. fermentum* L930BB (D), *B. animalis* subsp. *animalis* IM386 (E) and *L. plantarum* WCFS1 (F). NS – non-stimulated cells, CPT – cells treated with camptothecin, HT – heat treated cells.

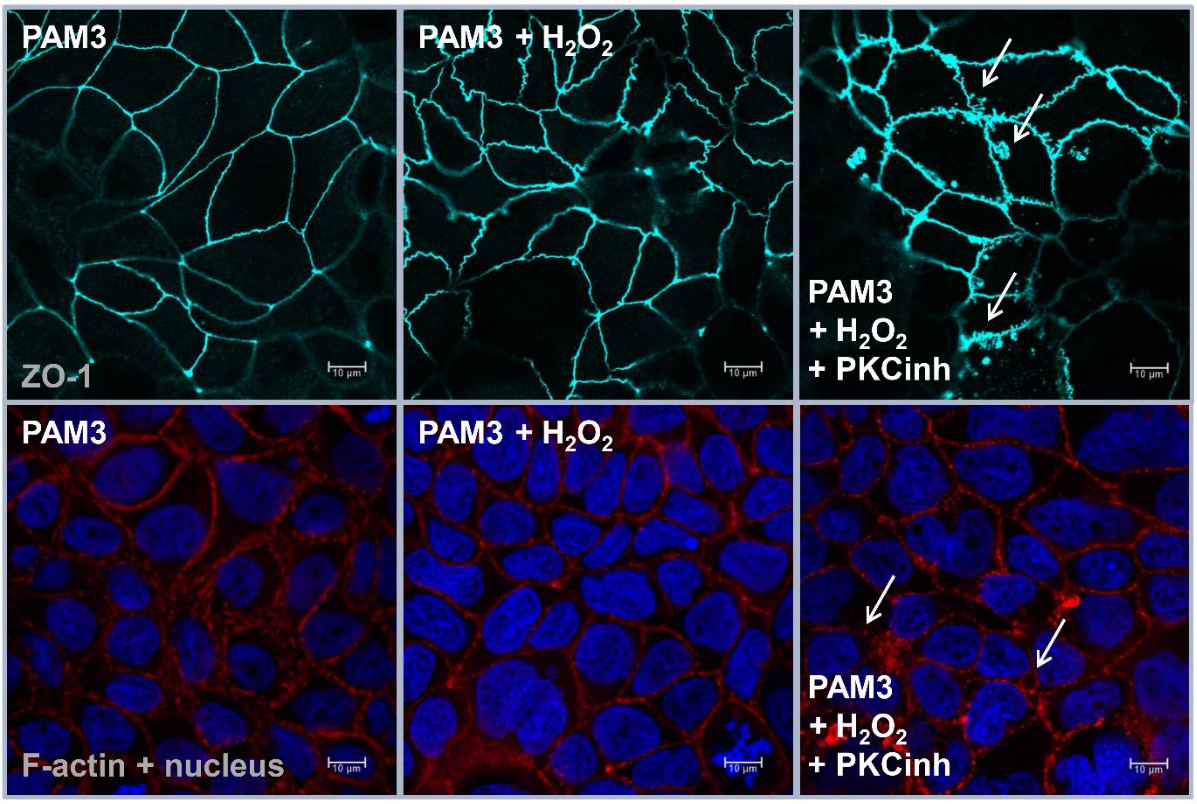

Figure S3: PKC-dependent impact of synthetic TLR2 ligand – PAM3 on rearrangement of ZO-1 and actin. Distribution of ZO-1 (blue) and F-actin (red) in untreated and with H_2O_2 treated Caco-2 cell line after stimulation with PAM3. Arrows indicate internalization of ZO-1 and interruptions in the actin ring. PKCinh – protein kinase C inhibitor Gö6983.

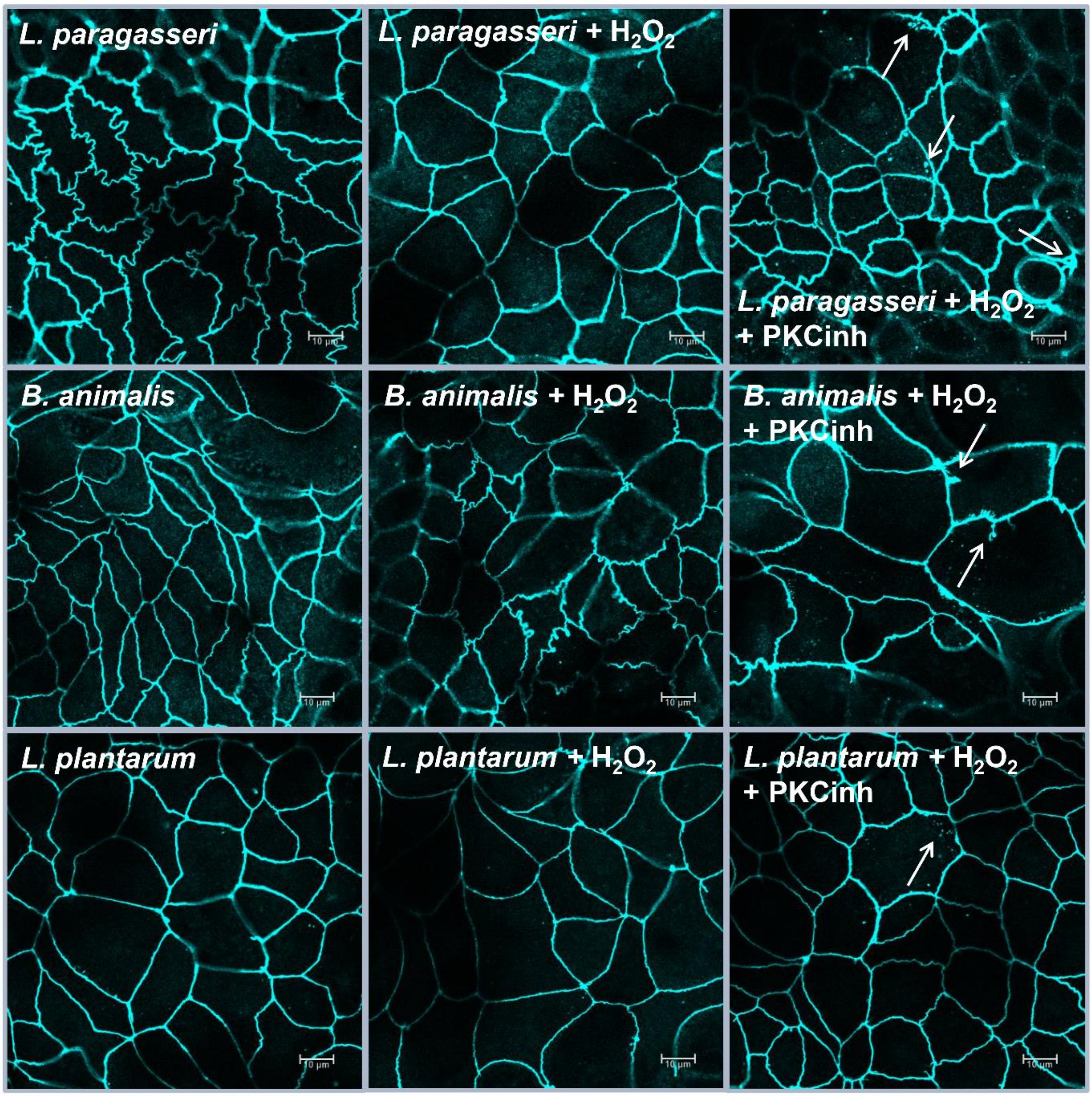

Figure S4: PKC-dependent impact of selected probiotic strains on rearrangement of tight junction protein ZO-1 (blue). Distribution of ZO-1 in untreated and with H_2O_2 treated Caco-2 cell line after probiotic stimulation. Arrows indicate internalization of ZO-1. Presented strains: *L. paragasseri* K7, *B. animalis* subsp. *animalis* IM386, *L. plantarum* WCFS1. PKCinh – protein kinase C inhibitor Gö6983.

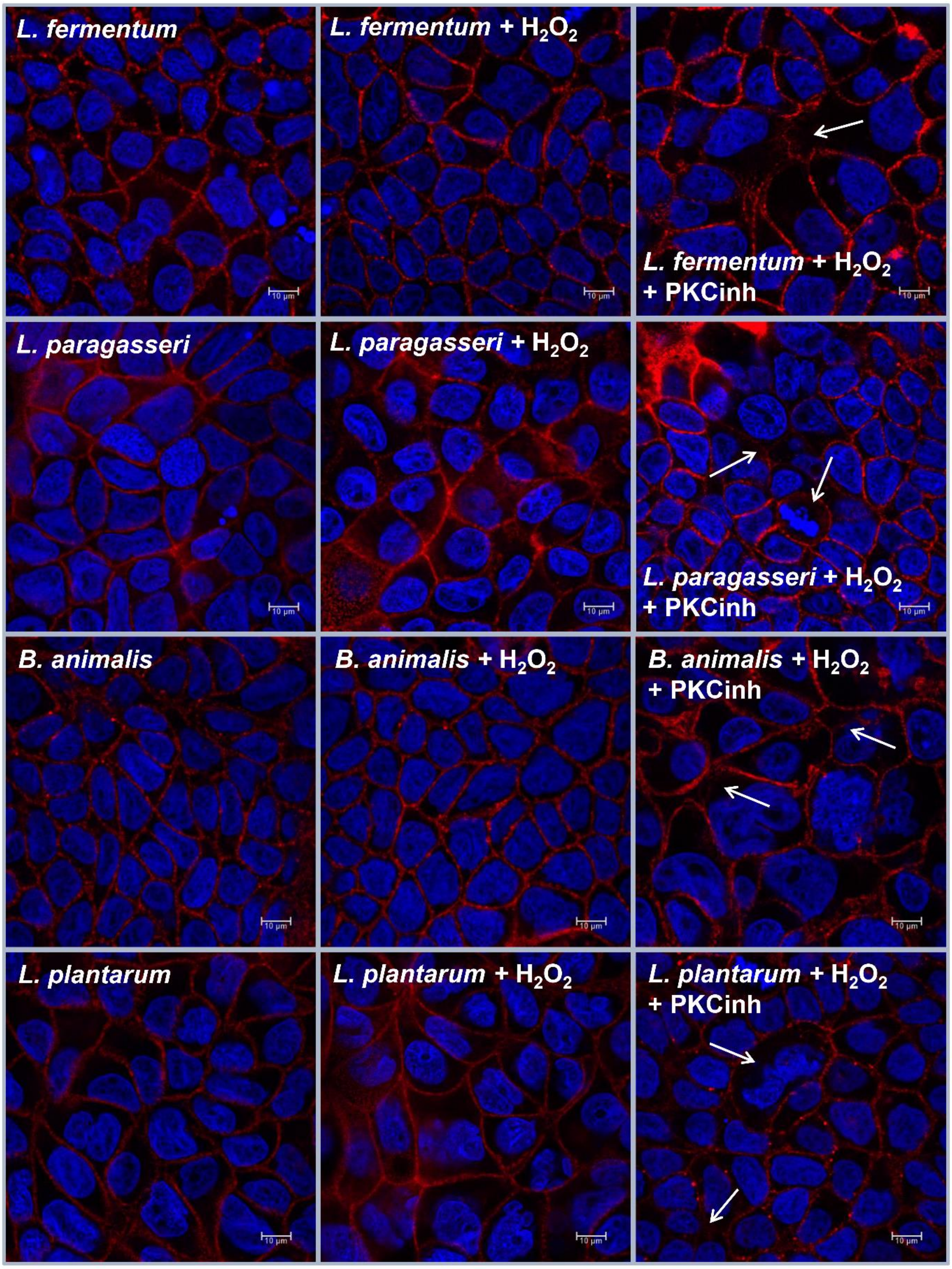

Figure S5: PKC-dependent impact of selected probiotic strains on rearrangement of F-actin. Distribution of F-actin (red) in untreated and with H_2O_2 treated Caco-2 cell line after probiotic stimulation, stained also for nucleus. Arrows indicate interruptions in the actin ring or dead cells. Used strains: *L. paragasseri* K7, *L. fermentum* L930BB, *B. animalis* subsp. *animalis* IM386, *L. plantarum* WCFS1. PKCinh – protein kinase C inhibitor Gö6983.

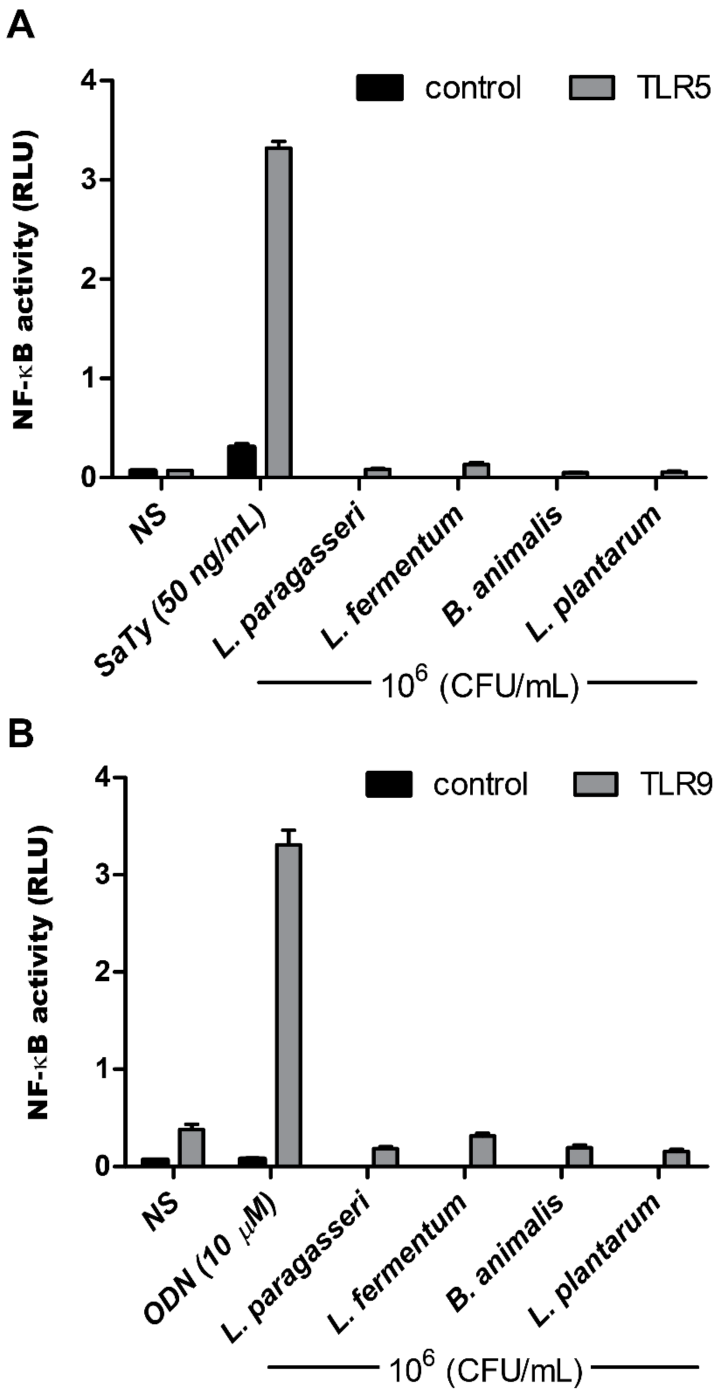

Figure S6: Evaluation of the transcription factor NF-κB activity after stimulation with selected probiotic strains through TLR5 and TLR9. TLR5 stimulation (A), TLR9 stimulation (B). The experiment was conducted in HEK293 cell line. Used strains: *L. paragasseri* K7, *L. fermentum* L930BB, *B. animalis* subsp. *animalis* IM386, *L. plantarum* WCFS1. Mean values ± standard deviation are shown. NS – non-stimulated cells, ODN – CpG-ODN 2006 Biotin, RLU – relative luciferase units, SaTy – *S. typhimurium* flagellin.
